# The substance of process

**DOI:** 10.15252/embr.202255480

**Published:** 2022-07-11

**Authors:** Veerle Horsting, Steffanie Hartjes

**Affiliations:** ^1^ Biomedical Science Programme, Faculty of Health, Medicine and Life Sciences Maastricht University Maastricht The Netherlands

**Keywords:** History & Philosophy of Science

## Abstract

A comment on “Everything flows. A process perspective on life” by Johannes Jaeger & Nick Monk.

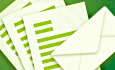

In the article “Everything Flows, A process perspective on life,” Johannes Jaeger and Nick Monk discuss substance‐ and process‐based views as complementary ways to explain reality: A substance is “anything that is truly universally and eternally unchangeable,” whereas a process is the “sequence of interconnected occurrences or events.” (Jaeger & Monk, 2015) Yet, a substance can also be described as material or matter with a definite chemical composition and distinct properties. A definite composition and distinct property, however, are not by definition eternally unchangeable. The notion that substances are static things or entities is true in the sense of their characteristics, and thus meaning, nonetheless, its function can be dynamic. Jaeger and Monk state that: “[t]hings that do not interact with something else are inert and thus irrelevant” and therefore reckon that a process “provides a richer and more natural picture of reality.” Although it is due to the interaction between the substances that processes carry this dynamic aspect, it is in fact the substances within the process that bear the feature of changing—for instance, from an inactive to an active form.

According to our interpretation, Jaeger and Monk aim to shift the focus from a substance to a process‐based view, rather than viewing them as concepts that are equally important to understand the bigger picture. They argue that the focus on substance has become unnecessarily limiting and is now impeding conceptual advances in science. They explain that “becoming is more important than being” and that “the traditional substance‐based stance struggles to explain—or even denies—the phenomena of agency, novelty or free will.” This particular view is shared by the book *The process genre* by Salomé Aguilera Skvirsky (2020), who explains process as a “representation of chronologically ordered steps in which some form of labor results in a finished product” and then goes on to illustrate with the examples of film, cookbooks, instruction videos, and so on how this plays a vital role in everyday life (Skvirsky, 2020). However, a structural foundation of substances can be found behind the processual representation. We think that by moving the focus from the substance‐ to a process‐based concept, the importance of the substance is undermined. In this way, a crucial component of the description is lacking. A process and a substance are not in contradiction with each other, but rather go hand in hand.

Furthermore, Jaeger and Monk claim that substances do not have any effect without a process while a process can exist separately from substances. They use examples of processes such as a thunderstorm, a burning flame, people's thoughts, a headache, and disease to demonstrate that these processes are no “things” and therefore exist without substance. The opposite is equally true: Processes cannot exist without substances. If it is not known what entities are involved and how these function, it is impossible to understand the biological phenomena mentioned above. The afore mentioned processes (thunderstorm, burning flame etc.) cannot be explained without knowing the meaning of the individual entities (air, plasma, neurotransmitters, pain receptors, and viral particles, respectively). This shows that in science, a process is as meaningful as a substance and neither should be neglected.

In “Freedom's Laboratory,” Audra Wolfe (2018) explains how the study of individual substances such as chromosomes and genes in *Drosophila* gave rise to discoveries of bigger processes such as heredity and new perspectives on evolution. Without the study of the smaller substances, this would have never been possible. *Vice versa*, processual, statistical studies of heredity offered valuable leads and ultimately resulted in the identification of the substance that allows the process (Watson & Crick, 1953).

We agree with Jaeger and Monk that process‐based thinking is unfortunately often secondary to substance‐based thinking in science, and we certainly believe that the gap between the two should be closed. However, we do not agree that the process‐based way of thinking should overrule the substance‐based perspective. We believe that understanding processes can never be complete without understanding the individual substances within. Nevertheless, we also believe that it is necessary to understand how these substances interact with each other and form a process. Drawing attention to the importance of process‐based research should therefore not come at the expense of the importance of substance‐based research as there is still so much to be explored in this area. Substances and processes both deserve equal credit in science.

## Disclosure and competing interests statement

The authors declare that they have no conflict of interest.
